# Feasibility and impact of laparoscopic sleeve gastrectomy after renal transplantation on comorbidities, graft function and quality of life

**DOI:** 10.1186/s12893-021-01138-x

**Published:** 2021-05-04

**Authors:** Naif A. AlEnazi, Khaled S. Ahmad, Ilham A. Elsamahy, Mohamed S. Essa

**Affiliations:** 1 Department of General Surgery, Ad Diriyah Hospital, Ar Rihab, Riyadh, Saudi Arabia; 2 Department of Anesthesia, Islamic Center for Heart Diseases and Cardiac Surgeries, Faculty of Medicine, El-Azhar University, Cairo, Egypt; 3 Department of General Surgery, Faculty of Medicine, Benha University Hospital, Benha University, Benha, Egypt

**Keywords:** Renal transplantation, Sleeve gastrectomy, Hypertension, Dyslipidemia, Graft function

## Abstract

**Background:**

The aim of this study is to clarify the feasibility and effect of laparoscopic sleeve gastrectomy (LSG) on comorbidities, graft function and quality of life in patients who underwent renal transplantation (RT).

**Methods:**

This is a retrospective review of five patients who underwent LSG after RT. Demographic data, anthropometric parameters, the effect on comorbidities, postoperative course, immunosuppressive medications, causes of RT, renal function, the survival of graft, and quality of life after SG in obese patients with a history of RT were assessed using BAROS–Moorhead–Ardelt survey

**Results:**

From September 2015 to September 2019, 5 renal transplant patients underwent LSG; three female, and two male. Median body mass index (BMI) decreased from 42.17 kg/m^2^ (range 36–55) before surgery to 28.16 kg/m^2^ (range 25–42) after surgery. Improvement in blood pressure, triglyceride, and cholesterol levels was observed, and all cases were able to decrease their medications. Insulin was stopped and replaced with linagliptin in all diabetic patients. Graft function improved, and proteinuria level decreased in all cases. All patients reported to have an excellent quality of life.

**Conclusion:**

LSG showed excellent outcomes in this high-risk group of patients regarding comorbidities, graft function and quality of life

## Background

Obesity has a strong relationship with hypertension (HTN) and type 2 diabetes (T2D), which are essential risk factors that lead to end-stage renal disease (ESRD). Furthermore, HTN and T2D are the most common causes of chronic kidney disease (CKD). It is noted that CKD is continually increasing at a rate of 7% per year. Interestingly, BMI has increased in CKD patients undergoing dialysis and also in patients on the waiting list for RT [1–6]. Several centers consider transplantation is contraindicated in morbidly obese patients because it is associated with a high rate of morbidity [7, 8].

Weight gain and morbid obesity following renal transplantation are considered a major health concern [9]. Multiple factors may provoke post-renal transplant weight gain, including immunosuppressant, hormonal changes, and lack of physical activity [10, 11]. Obesity has a significant burden on both pre- and post-transplantation patients, including a high risk of graft loss and delayed graft function [12]. Obesity can negatively influence wound repair and increase the risk of postoperative hernias [13]. Also, it is associated with shortening patient survival and decreased quality of life [12, 13]. Bariatric surgery, especially LSG, is effective in reducing weight and obesity-related complications among post-renal transplantation population [14, 15]. Nonetheless, the efficacy of LSG for post-renal transplantation patients has not been thoroughly investigated. This case series retrospectively reviews and highlights the outcomes of five patients who underwent LSG following a kidney transplant.

## Methods

This study involves 5 patients who underwent LSG post-RT from September 2015 to September 2019. The data of the patients were assessed retrospectively after approval by institutional review board (IRB) of Saudi German Hospital (No.17918). Data regarding patients’ age, gender, medical history, operative time, timing from RT to LSG and BMI before RT and LSG and after LSG were assessed. Graft function and survival (defined as no clinical or laboratory evidence of graft rejection) associated with post-RT sleeve gastrectomy for obese patients were assessed. Both graft function and survival were assessed by serum creatinine level, urine protein level, and urine output every 3, 6, 9, 12 months after surgery, then every year during a follow-up period. BAROS–Moorhead–Ardelt score was used for the evaluation of the quality of life before surgery and 1 year after surgery. This score assesses the percentage of excess weight loss (%EWL), comorbidities resolution, and quality of life score from 1 to 9. BAROS–Moorhead–Ardelt score is: failure (≤ 1), fair enough (1–3), good results (˃3–5), very good (˃5–7), and excellent (> 7–9) [16]. %EWL calculated by dividing the number of kilograms lost by the number of kilograms in excess body weight (EBW) of the patient. %EWL measured 3, 6, 9, 12 months after surgery then yearly.

### Exclusion criteria

The following patients were excluded from the study: (1) age ˂ 18 years old, (2) Simultaneous non-renal transplant, (3) Previous non-renal transplant, (4) Primary non-functioning graft AND (5) Data regarding BMI at the time of transplant not available.

### Outcomes

Primary outcome includes impact of LSG on %EWL and graft function while secondary outcome is impact of LSG on comorbidities and medications.

### Follow up

Postoperative clinical follow-up was done every 3 months in the first year, then yearly, and included, history and physical examination, calculation of %EWL in addition to checking of creatinine level and urine protein level.

### Surgical technique

Under direct vision, 12-mm optical trocar was inserted for the camera in the midline supraumbilical position. After that, two other ports were inserted (one 5 mm on the left hypochondrium and one 5 mm on the right hypochondrium) in addition to a 10-mm trocar that was inserted on the left flank. PretzelFlex liver retractor was introduced in the epigastric region. Dissection of the greater omentum and short gastric vessels from the gastric greater curvature till the left crus of the diaphragm using Harmonic scalpel. A 42-Fr bougiewas inserted. Gastric divison started approximately 4–6 cm from the pylorus and parallel to the lesser curvature of the stomach and extended vertically toward His angle using between 4 and 5 linear staplers such as Echelon 60. Black (4.2 mm), green (4.1 mm), gold (3.8 mm), or blue (3.5 mm) cartridges were applied based on the gastric wall thickness. The reinforcement of the staple line was done using an absorbable suture in running fashion. The specimen was retrieved in a plastic bag.

## Results

**Fig. 1:**
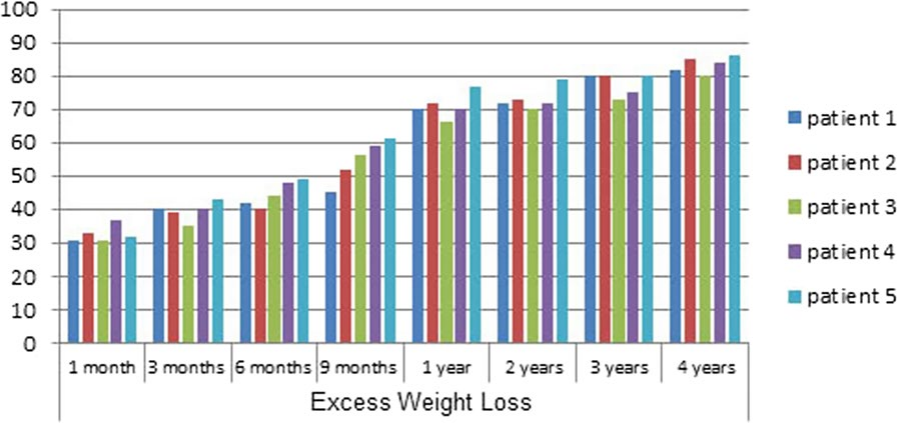
%EWL

This study included five patients who underwent post-RT laparoscopic sleeve gastrectomy between September 2015 and September 2019. There were three females and two males. The median BMI before RT was 29.24 kg/m^2^ (range 22–41) and before SG was 42.17 kg/m^2^ (range 36–55). Demographic data, comorbidities, timing from RT to LSG, and operative duration are shown in (Table 1). Anthropometric parameters pre-RT, pre-SG, and post-SG are shown in (Table 2). The median follow-up period was 24 months (range 7–48). At that time, the median BMI was 28.45 kg/m^2^ (range 25–42), and %EWL was 56.4% (range 31–86) (Fig. 1). Associated comorbidities were HTN and dyslipidemia (DSL) in all patients. Three patients had T2D, and 2 had gout.

**Table 1:** Demographic data, comorbidities, timing fromRT to LSG and operative time

Number	Age (years)	Sex	Medical history	Time from RT to LSG (years)	Operative time (min)
Patient 1	40	Male	HTN, Dyslipidemia, Gout	6	62
Patient 2	52	Female	HTN, DM, dyslipidemia	6	58
Patient 3	33	Female	HTN, Dyslipidemia, DM	4	65
Patient 4	38	Male	HTN, dyslipidemia	3	50
Patient 5	36	Female	HTN, DM, dyslipidemia	9	70

*HTN* hypertension, *DM* diabetes mellitus, *LSG* laparoscopic sleeve gastrectomy, *BMI* body mass index, *min* minute

**Table 2:** Anthropometric parameters

Anthropometric data	Median (range)
BMI before RT (kg/m^2^)	29.24 (22–41)
BMI before LSG (kg/m^2^)	42.17 (36–55)
Weight before LSG (kg)	112 (92–130)
BMI after LSG (kg/m^2^)	28.45 (25–42)
Follow-up after LSG (months)	24 (7–48)

*RT *renal transplantation, *LSG* laparoscopic sleeve gastrectomy, *BMI b*ody mass index

Regarding comorbidities, the results were as follows: improvement in blood pressure occurred in of patients from 145 to 155 /89–100 mmHg LSG to 120–130/70–90 mmHg after LSG (*p* = 0.263). Also, a reduction in cholesterol level has been observed; the median cholesterol level was reduced from 332 mg/dl (range 315–356) before the surgery to 216 mg/dl (range 192–220) after surgery (p = 0.247). The median Triglycerides level reduced from 200 mg/dl (range 195–240) to 155 mg/dl (range 125–170) (*p* = 0.196) (Table 3). Regarding diabetes mellitus (DM), 3 patients were T2D. All were receiving Novomix insulin (2 patients received Novomix 40U/day, and 1 patient Novomix 60U/day) before surgery. After surgery, linagliptin (5 mg/day) replace insulin in all cases. The median A1C reduced from 8 before LSG to 6.2 after LSG at 48 months follow-up period (Table 4).

**Table 3:** Evolution in cholesterol, triglyceride and blood pressure post-SG

Comorbidities		Patient 1	Patient 2	Patient 3	Patient 4	Patient 5
Cholesterol (mg/dl) (median)	Pre-SG	345	328	332	315	356
	Post-SG	210	205	216	192	220
Triglycerides (mg/dl) (median)	Pre-SG	240	220	200	197	195
	Post-SG	170	160	150	155	125
Blood pressure (mmHg)	Pre-SG	150–155/90–94	145–150/95–100	149–155/90–95-	150–155/89–95	145–150/95–100
	Post-SG	120–125/80–85	125–130/80–90	125–130/75–80	120–130/80–85	125–130/70–78

*SG* sleeve gastrectomy

**Table 4:** Improvement in A1C and medications after SG

Patients	Type of DM	Timing	A1C	Medications
Patient 2	T2D	Pre-LSG	8.6	Biphasic insulin 70:30 (Novomix) 40 U daily
		Post-LSG	6.4	Linagliptin 5 mg/day
Patient 3	T2D	Pre-LSG	8	Biphasic insulin 70:30 (Novomix) 40 U daily
		Post-LSG	6.2	Linagliptin 5 mg/day
Patient 5	T2D	Pre-LSG	9	Biphasic insulin 70:30 (Novomix) 60 U daily
		Post-LSG	6.7	Linagliptin 5 mg/day

*T2D* diabetes mellitus type2, *A1C* glycosylated hemoglobin, *LSG* laparoscopic sleeve gastrectomy

Before RT, all patients were on hemodialysis. Post-RT, all cases received immunosuppressive medications: steroids and cyclosporine were given for all patients. In addition, 4 patients received mycophenolate, 1 patient azathioprine, 1 patient tacrolimus, and 1 patient everolimus. Indications for RT were systemic lupus erythematosus (SLE), HTN, DM, interstitial nephritis and glomerulonephritis. The median time between RT and LSG was 4 years (range 3–9). The median operative duration for LSG was 65 min (range 50–70). The median length of hospital stay was 5 days (range 4–6). Postoperative recovery was uneventful. There were no complications or mortality after surgery in this series.

**Fig. 2:**
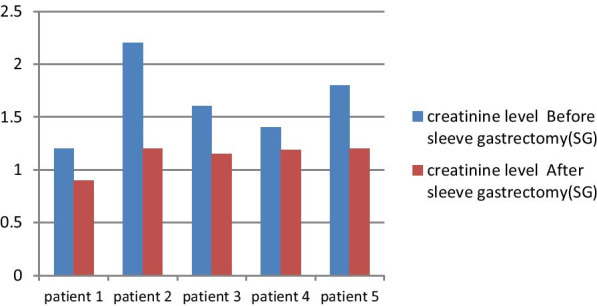
Graft function (creatinine level) before and after LSG

The median creatinine level was 1.6 mg/dl (range 1.2–2.2) preoperatively, after LSG, it decreased to 1.18 mg/dl (range 0.9–1.5) (*p* = 0.253), creatinine levels improved in all cases (Fig. 2). The median protein level in urine decreased from 30 mg/dl (range 10–37) before the surgery to 12 mg/dl (range 8–18) after surgery (*p* = 0.261). As creatinine levels, protein levels in urine decreased in all patients post-LSG.

All patients reported an excellent quality of life after surgery, the median BAROS–Moorhead–Ardelt score before LSG was 1 (range 1–3) and after LSG was 7 (range 7–9). They were satisfied with the results after surgery.

## Discussion

Weight gain and obesity are common conditions affecting around half of the post-kidney transplant patients. Post-grafting weight gain is associated with the incidence of hypertension, diabetes mellitus, hyperlipidemia, graft rejection, and decreased graft survival [4, 17]. Curran et al., reported that patients with BMI ≥ 30 kg/m^2^ at the time of transplant was associated with high risks of delayed graft function (DGF) while patients with a BMI ≥ 35 kg/m^2^ were at greater risks of biopsy-proven acute graft rejection (BPAR) and all-cause graft failure [18]. Thus, effective weight reduction procedures are essential to preclude these complications. Bariatric surgery is an effective ingrained management choice for morbid obesity in particular cases [14]. Of note, LSG, compared to other bariatric operations, has promising efficacy and reliability [14, 15].

The American Society of Metabolic and Bariatric Surgery (ASMBS) has approved the LSG as the first-stage procedure for sustained weight reduction in 2012 [19]. Previous reports have indicated that LSG has several salutary features including short operative time, short hospital stay, good short-term outcomes regarding %EWL and disappearance and/or improvement of comorbidities. In addition, and in case of failure, it can be converted to another bariatric procedure with low morbidity and mortality. It is considered a non-complex procedure compared to gastric bypass. Therefore, these advantages are of the greatest value for patients who are primarily liable to the incidence of complications [19–26].

Furthermore, LSG has been considered as the first-choice intervention for pre-transplant candidates, as well as the post-liver transplant population [27, 28]. Thus, we believe that LSG is the proper decision for morbid obesity and weight reduction in post-renal transplant patients. A major advantage of LSG is that it does not interfere with the absorption of immunosuppressant drugs [29]. These drugs play an intrinsic role in preventing acute graft rejection and lengthening the graft survival. In contrast, malabsorptive procedures such as Roux-en-Y gastric bypass (GB) and biliopancreatic diversion may manipulate the pharmacokinetics of immunosuppressant drugs. This has been stated in Golomb et al. study, whereas two patients required a higher dose of tacrolimus while the dose was decreased in one patient [30].

LSG also improves transplant eligibility by decreasing BMI in renal transplant candidates and prevent development of new onset DM after transplantation (NODAT). Kim Y et al., reported 20 patients underwent LSG before RT and compared with similar-BMI recipients who did not underwent LSG using signed-rank and McNemar's tests. In comparison to patients without LSG, Patients with post-LSG RT had lower rates of DGF (5% vs 20%) and graft dysfunction –related readmissions (10% vs 27.5%) (p < 0.05 for both) in addition to no patients developed NODAT. These results suggest that LSG for obese patients with ESRD result in improvement in transplant candidacy and post-transplantation outcomes [31].

Golomb et al. performed LSG for 10 post-renal transplant patients and concluded a successful procedure in 80% of cases without graft rejection or dysfunction [17]. Also, urinary protein and serum creatinine markedly decline following LSG, which is consistent with our findings as well as previously published reports [30–32]. In another recent study, creatinine levels were normalized following bariatric surgery, and kidney function slightly improved 12 months after surgery. The exact value and impact of this improvement have not yet clearly understood [33]. However, proteinuria has been recognized as a poor prognostic marker and risk factor of mortality among kidney transplant patients [34]. Thus, it is plausible that this improvement might have a long-term effect on graft function and survival [30].

This retrospective case series had several limitations. First, it included only five subjects that encountered the generalization and reliability of LSG as a standard measure for weight reduction in kidney transplant patients. Second, the follow-up extended to 4 years; however, it may not be sufficient to examine graft function and graft survival accurately. Therefore, our findings need to be further advocated by large-scale random studies with extended follow-up. Also, future studies are recommended to compare their inferences with kidney transplant patients who were not subjected to bariatric surgery.

## Conclusion

Our study showed that there were no pre- or postoperative complications reported. Diabetes and hypertension were managed, and the patients discontinued the medications after bariatric surgery. Urinary proteins and lipid levels were settled, graft function was improved, and all patients reported enhanced quality of life.

## Data Availability

All data generated or analyzed during this study are included in this published article and it is available from the corresponding author on reasonable request.

## Abbreviations

RT: Renal transplantation
LSG: Laparoscopic sleeve gastrectomy
SG: Sleeve gastrectomy
HTN: Hypertension
DSL: Dyslipidemia
BMI: Body mass index
ESRD: End stage renal disease
CKD: Chronic kidney disease
DM: Diabetes mellitus
T2D: Diabetes mellitus type 2
A1C: Glycosylated hemoglobin
GB: Gastric bypass
ASMBS: American Society of Metabolic and Bariatric Surgery
DGF: Delayed graft function
BPAR: Biopsy proven acute rejection
NODAT: New onset diabetes mellitus after transplantation
